# Preclinical Evaluation of Polymeric Nanocomposite Containing Pregabalin for Sustained Release as Potential Therapy for Neuropathic Pain

**DOI:** 10.3390/polym13213837

**Published:** 2021-11-06

**Authors:** Rafaela Figueiredo Rodrigues, Juliana Barbosa Nunes, Sandra Barbosa Neder Agostini, Paloma Freitas dos Santos, Juliana Cancino-Bernardi, Rodrigo Vicentino Placido, Thamyris Reis Moraes, Jennifer Tavares Jacon Freitas, Gislaine Ribeiro Pereira, Flávia Chiva Carvalho, Giovane Galdino, Vanessa Bergamin Boralli

**Affiliations:** 1 Faculdade de Ciências Farmacêuticas, Universidade Federal de Alfenas (UNIFAL-MG), Alfenas 371300-001, Brazil; sbneder@gmail.com (S.B.N.A.); paloma.nut13@gmail.com (P.F.d.S.); rodrigov_placido@hotmail.com (R.V.P.); jenniferjaconfreitas@gmail.com (J.T.J.F.); gislaine.pereira@unifal-mg.edu.br (G.R.P.); flaviachiva@gmail.com (F.C.C.); 2 Instituto de Ciências Médicas, Universidade de São Paulo (USP), São Paulo 01246-903, Brazil; juliana_bnunes@yahoo.com.br; 3 Instituto de Física de São Carlos, Universidade de São Paulo (USP), São Carlos 13566-590, Brazil; jcancinobernardi@gmail.com; 4 Instituto de Ciências da Motricidade, Universidade Federal de Alfenas (UNIFAL-MG), Alfenas 37133-840, Brazil; thamoraes@yahoo.com.br (T.R.M.); giovane.souza@yahoo.com.br (G.G.)

**Keywords:** neuropathy, polymeric nanoparticles, preclinical investigation, pharmacokinetics of pregabalin, antinociceptive effect, induced sleep

## Abstract

This study offers a novel oral pregabalin (PG)-loaded drug delivery system based on chitosan and hypromellose phthalate-based polymeric nanocomposite in order to treat neuropathic pain (PG-PN). PG-PN has a particle size of 432 ± 20 nm, a polydispersity index of 0.238 ± 0.001, a zeta potential of +19.0 ± 0.9 mV, a pH of 5.7 ± 0.06, and a spherical shape. Thermal and infrared spectroscopy confirmed nanocomposite generation. PG-PN pharmacokinetics was studied after a single oral dose in male Wistar rats. PG-PN showed greater distribution and clearance than free PG. The antinociceptive effect of PG-PN in neuropathic pain rats was tested by using the chronic constriction injury model. The parameter investigated was the mechanical nociceptive threshold measured by the von Frey filaments test; PG-PN showed a longer antinociceptive effect than free PG. The rota-rod and barbiturate sleep induction procedures were used to determine adverse effects; the criteria included motor deficit and sedative effects. PG-PN and free PG had plenty of motors. PG-PN exhibited a less sedative effect than free PG. By prolonging the antinociceptive effect and decreasing the unfavorable effects, polymeric nanocomposites with pregabalin have shown promise in treating neuropathic pain.

## 1. Introduction

Pregabalin (PG) (S-[+]-3-isobutyl GABA or (S)-3-aminomethyl-5-methylhexanoic acid) is an anticonvulsant, antihyperalgesic, and anxiolytic drug that acts by binding to the alpha-2-delta-1 proteins of voltage-dependent calcium channels in the Central Nervous System (CNS), reducing the release of excitatory neurotransmitters [1]. PG is one of the first-choice medicines for the treatment of neuropathic pain that has been authorized by the FDA [2]. Due to its short half-life, PG is sold as an instant release (IR) tablet, with a daily dosage of 150 to 600 mg split into two or three administrations [1,3]. In addition, sleepiness, dizziness, and loss of consciousness are common adverse effects of PG [1]. According to studies, around 15% of patients using pregabalin for neuropathic pain discontinue their therapy due to adverse effects, even when the dosages are tolerable [4,5]. PG is a class I molecule with good solubility and permeability, according to the Biopharmaceutical Classification System (BSC) [6], indicating that it has no physicochemical issues that would require a change in pharmaceutical form. As neuropathic pain is persistent and needs lengthy therapy, these two variables (short half-life and adverse effects) might be a barrier for appropriate treatment compliance [3].

The FDA has approved the commercialization of PG extended-release coated tablets (Lyrica CR^®^) as a means to avoid the discomfort of numerous doses [7]. Even if formulation development was successful, three flaws may be addressed in terms of once-daily dosing: (a) controlled-release (CR) tablets must be taken after an evening meal; (b) this evening meal must be hypercaloric (800 to 1000 kcal) in order to achieve the same level of absorption as PG IR; (c) the CR formulation has essentially the same side effects as IR tablets, with similar user incidences [8].

Some studies have proposed modified-release pharmaceutical formulations containing PG for once-daily administration, such as transdermal delivery of PG [9]; PG microspheres [10,11,12]; PG formulations with longer stomach duration [6,13,14,15,16,17,18,19,20]; and PG suppository [21]. The treatment of neuropathic pain has been the focus of several of these formulations.

These formulations, which were created for once-daily PG delivery, demonstrated that controlled release methods may be used to improve treatment adherence. However, none of the formulations that were previously provided assessed the reduction in adverse effects. This is an essential aspect to address because side effects are linked to treatment adherence [4,5]. Furthermore, the majority of studies have not evaluated the formulation’s effectiveness in terms of pain/nociception reduction. As a result, there is a significant research gap as well as an opportunity to enhance therapy in experimental studies with the potential to be used in clinical practice.

Many investigations have been conducted recently on the use of natural polysaccharides (e.g., alginate, chitosan, hyaluronic acid, cellulose, and starch) for various biological, biomedical, functional food, and tissue engineering applications due to their biocompatibility and biodegradability [22,23,24,25]. These natural polysaccharides have been widely used as carriers for the delivery of various therapeutic molecules (e.g., proteins, peptides, and drugs), mainly for anti-cancer therapies, diabetes, and other chronic diseases [24,26,27]. Polymeric nanoparticles are one form of drug delivery system that can be based on natural polymers such as chitosan (CS) and hydroxypropylmethylcellulose phthalate (HPMCP) or hypromellose phthalate, and they are advantageous due to desirable properties such as stability, safety, non-toxicity, hydrophilicity, and biodegradability in addition to being abundant in nature and having low processing costs [28,29]. Drug carrier nanoparticles can be used as an alternative pharmaceutical form because they allow a significant increase in the drug’s bioavailability in CNS, increased specificity of the drug at its site of action, increased distribution in the body, dose reduction, and reduced adverse effects [30,31].

In a model of chronic sciatic nerve constriction, we aimed to develop a PG-loaded polymeric nanocomposite formulation utilizing CS and HPMCP that would extend antinociceptive effects, improve nociception perception, and enable administration once daily without generating adverse effects.

## 2. Materials and Methods

### 2.1. Materials

Pregabalin was purchased from Pfizer^®^ (Karlsrufe, Germany). Low viscosity shrimp chitosan (150 kDa) was from Sigma-Aldrich^®^ (Saint Louis, MS, USA). Hypromellose phthalate (HPMCP, type HP-55) was kindly donated by Shin-Etsu Chemical Co.^®^ (Tokyo, Japan). Ketamine hydrochloride 10% injectable was purchased from Linavet^®^ (Rio de Janeiro, Brazil). Injectable xylazine was purchased from Hertape Calier Saúde Animal S.A. (Juataba, Brazil). Isoflurane and sodium thiopental were purchased from Cristália (Itapira, Brazil). Injectable sodium heparin was purchased from Blau Farmacêutica S.A. (São Paulo, Brazil). Metformin was purchased from USP Reference Standard (Betheseda, Rockville, MD, USA). Sodium phosphate dibasic heptahydrate PA and sodium phosphate tribasic dodecahydrate PA were purchased from Vetec^®^ (Duque de Caxias, Brazil). Sodium phosphate monobasic monohydrate PA was purchased from Proquimios^®^ (Rio de Janeiro, Brazil). Sodium hydroxide PA was purchased from Sigma-Aldrich^®^ (Saint Louis, MS, USA). Glacial acetic acid PA was purchased from Dinâmica Química Contemporânea Ltd.a. (Indaiatuba, Brazil). Hydrochloric acid 37% PA and formic acid were purchased from Alphatec^®^ (Macaé, Brazil). Acetonitrile and Methanol HPLC grade were purchased from J.T. Baker^®^ (Phillipsburg, NJ, USA). Ammonium formate HPLC was purchased from Sigma-Aldrich^®^ (Saint Louis, MS, USA).

### 2.2. Obtaining Pregabalin-Loaded Polymeric Nanocomposite (PG-PN)

Polymeric nanocomposites were prepared by ionic crosslinking of CS dispersion with HPMCP aqueous solution, according to the ionotropic gelation method [32].

The solution of CS was prepared by the dispersion of 4 mg/mL of chitosan in acetic acid solution (0.1 M, pH = 5.5) at room temperature (24 °C) and mechanical stirring at 800 rpm (Velp Scientifica^®^, Usmate Velate, Italy). PG solution, made of PG (8 mg/mL), was dissolved in a sodium phosphate buffer solution (0.2 M, pH = 6.0) at room temperature. The solution of HPMCP was prepared by dissolving HPMCP (2 mg/mL) in sodium hydroxide solution (0.1 M, pH = 5.5) at room temperature, and mechanical stirring was conducted at 800 rpm.

The PG solution was added dropwise to CS dispersion (3.18 PG: 3 QS % *m/m*) under mechanical stirring at 800 rpm at room temperature. Then, the HPMCP solution (3 QS: 1 HPMCP, % *m/m*) was added dropwise into the mixture under mechanical stirring at 800 rpm at room temperature. The nanocomposite dispersion was kept under mechanical stirring at 800 rpm for 30 min at room temperature (24 °C). For dripping, an insulin syringe (100 U.I.) (Descarpack, São Paulo, Brazil) with a 26.5 g ½” needle attached (São Paulo, Descarpack, Brazil) was used for each solution at the speed of 100 drops per minute. The ratio of polymers (3 QS: 1 HPMCP, % *m/m*) was chosen based on a previous study by our research group [33]. The same method was used to make empty polymeric nanocomposites (EMP-PN) or PG-free by using CS solution, HPMCP solution, and 0.2 M sodium phosphate buffer pH = 6.0.

### 2.3. Experimental Animals

This study utilized conventional heterogeneous male Wistar rats (*Rattus norvegicus albinus*) weighing 220 to 250 g, 7 weeks old, and housed in groups of five per cage with shavings as bedding material under a 12 h light/dark cycle at a constant room temperature (24 ± 2 °C) and humidity (60%). Water and standard chow were available ad libitum. All experiments were carried out within the animals’ circadian cycle, respecting the light cycle, between 7 and 19 h. If animal manipulation was necessary after the light cycle, a 15-watt red lamp was used to illuminate the room. All procedures followed the Committee for Research and Ethical issues of the International Association for the Study of Pain [34], and the study was approved by the Ethics Committee on the Use of Animals of Universidade Federal de Alfenas, Brazil (protocol number 57/2016).

All animals were randomly assigned to an experimental group based on the treatment they received orally: water (animals treated with ultra-pure water–control), pregabalin (PG) (animals treated with free pregabalin), PG-PN (animals treated with polymeric nanocomposite containing pregabalin), and EMP-PN (animals treated with polymeric nanocomposite without pregabalin). For this, free PG was prepared by dissolving PG in distilled water. PG-PN and EMP-PN were prepared according to 2.2 item. All formulations were prepared shortly before use and administrated orally in a single dose by gavage (2.5 mL/kg). The dose was chosen based on the research group’s earlier investigations [35], which used a dose of 10 mg pregabalin/kg animal weight.

### 2.4. Characterization of PNs

Dynamic light scattering (DLS) and electrophoretic light scattering on a Nano Zs (Malvern Instruments^®^, Worcestershire, England) were used to assess particle size, polydispersity index (PDI), and zeta potential. After being kept in an ultrasonic bath for 2 min, all samples were examined without previous dilution. The tests were carried out in triplicate at 25 °C, with an attenuation value of 8 and a detection angle of 173°.

Nanoparticle tracking analysis (NTA) on the Nanosight NS 300 (Malvern Instruments^®^, Worcestershire, England) was also used to measure particle sizes. The samples have previously been diluted in distilled water (1:5000). Three measurements were made in sequence at 25 °C.

An Mpa-210 pH meter (Tecnopon^®^, Piracicaba, Brazil) was used to determine the pH without previous dilution.

The samples were diluted (1:100) in ultrapure water before being dripped onto previously cleaned silicon support, dried in a vacuum drying apparatus, and metalized with carbon for morphological evaluation. After that, the samples were examined by using a high-resolution field emission scanning electron microscope SEM-FEG JEOL^®^ JSM-7500F (Tokyo, Japan) with an energy dispersive spectroscopy (EDS) Ultra Dry model detector (Thermo Scientific^®^, Waltham, MA, USA).

The possible interactions between PG and the polymers in the nanostructured system were analyzed by Fourier transform infrared spectroscopy (FT-IR) (FT-IR Affinity^−1^, Shimadzu^®^, Tokyo, Japan) scanning at 4000 to 600 cm^−1^, with 64 scans at 4 cm^−1^ resolution, using the dropping technique on potassium bromide (KBr) pellets. The samples were concentrated 4 times; 3 drops of PG-PN or EMP-PN (15 µL each drop) were dropped onto a KBr pellet, with a 30 min interval between drops. After adding 3 drops, the KBr pellets were placed to dry in a desiccator containing silica under vacuum for 19 h. After drying, the KBr pellet was analyzed. The KBr pellets containing the solutions of the polymers and the drug were prepared and analyzed in the same manner.

A differential exploratory calorimeter DSC 3500 Sirius (Netzsch^®^, Selb, Germany) was used to analyze freeze-dried PN samples, calibrated with Indium, Tin, Bismuth, and Zinc standards, by placing the sample (5–7 mg) in a closed aluminum sample holder with a perforated lid under a dynamic nitrogen atmosphere (50 mL/min) with a heat flow of 10 °C/min.

Thermogravimetric measurements (TG) and Differential Thermal Analysis (DTA) were taken by using freeze-dried samples with masses ranging from 3.4 to 8.2 mg (depending on sample particle size) in aluminum sample holders and a heating rate of 10 °C/min in a simultaneous thermogravimetric TG/DTA 7300 module (Star^®^, Kyoto, Japan) calibrated with Indium standard. The tests were conducted at temperatures ranging from 30 to 550 °C under an inert atmosphere of nitrogen at a steady flow rate of 50 mL/min.

### 2.5. Pharmacokinetic Study

For the pharmacokinetic study, the rats were split into two groups (n = 12): PG and PG-PN. Before the treatments, each rat was cannulated in the jugular vein to collect blood and was housed in its cage [36]. Drugs were administered 12 h after cannulation, and 500 µL of blood samples was taken after 0.16, 0.5, 1, 1.5, 2.5, 4, 6, 8, 12, 24, 36, and 48 h, and the volume was reposed with sterile saline. After the last blood collection, the animals were euthanized by an excess of anesthetic (isoflurane 8%, inhalation route). The lack of vital signs and mucosal stains indicated death. In order to avoid interference with absorption, the animals received water ad libitum during the experiment and were fasted for at least six hours before and up to two hours following drug administration.

The samples were collected in heparinized tubes and centrifuged (2500× *g* for 10 min). The plasma was separated and stored at −70 °C for the pregabalin assay.

Pregabalin was measured by using high-performance liquid chromatography-mass spectrometry (LCMS-8030, Shimadzu^®^, Tokyo, Japan) in positive electrospray ionization (ESI) mode, with the following mass transitions monitored: Pregabalin was measured at 159.85 > 142.10; 159.85 > 97.20; and 159.85 > 83.20. Metformin was used as an internal standard (IS) and measured at 130.10 > 60.05; 130.10 > 71.05; and 130.10 > 83.20. The method was validated by using a pool of blank plasma (free of any chemical). The sample preparation for analysis included precipitation with acetonitrile (1:10 plasma: acetonitrile) and subsequent high-speed centrifugation (17,800× *g* for 10 min). After centrifugation, 900 microliters of the supernatant was collected and vacuum evaporated at 80 °C. The residue was resuspended in 200 microliters of mobile phase (the gradient’s initial concentration), and 50 microliters was chromatographically analyzed.

The mobile phase was acetonitrile: 2 mM ammonium formate solution pH 3.0 in a gradient flow (initial condition 70:30% *v*/*v*, maintained until 7 min, followed by a linear reduction of 1 min of the organic phase ratio to 50:50% *v*/*v*, which was maintained for 5 min to clean the column and followed by a linear return to the initial conditions for 1 min and stabilization of the column at the initial condition for 5 min) with a constant flow rate of 0.2 mL/min. Formic acid was used to adjust the pH of the ammonium formate solution. As a stationary phase, a BEH HILIC ACQUITY UPLC column (1.7 µm, 2.1 mm × 50 mm) was used with an oven set to 50 °C, a UV detector set at 190 nm, and a chromatographic analysis time of 19 min. The nebulizer gas flow was 1.5 L/min, the DL temperature was 250 °C, the heating block temperature was 400 °C, and the drying gas flow was 15 L/min in the mass spectrometer.

The method was validated according to the Food and Drug Administration’s (FDA) validation guidelines [37], with a detection threshold of 1.17 µg/mL of plasma. Aside from that, the technique was precise and accurate, with a linear range of 0.1–12.50 µg/mL of plasma.

Pharmacokinetic parameters were calculated based on plasma concentrations. In order to evaluate differences between the groups’ PG and PG-PN, bioavailability (measured by the area under the curve—AUC), distribution (represented by the volume of distribution—Vd), and elimination (expressed by the half-life—t_1/2_ and clearance) parameters were used.

The curve was constructed by using plasma concentration versus time (AUC^0-^^∞^) and estimated using the trapezoid technique [38]. The PKSolver add-in in Microsoft Excel^®^ was used to perform pharmacokinetic analysis [39].

### 2.6. Antinociceptive Effect Study

The sciatic nerve’s chronic constriction injury (CCI) was employed as a model for the induction of neuropathic pain [40]. In summary, animals were anesthetized with ketamine (90 mg/kg, intraperitoneal route—IP) and xylazine (10 mg/kg, IP), and the sciatic nerve was exposed and loosely ligated with 4-0 chronic gut thread at four sites with a 1 mm interval.

The rats were randomly assigned to one of four groups (n = 12 each group): water; PG; PG-PN; or EMP-PN. Each group consisted of six CCI rats (n = 6) and six sham rats (n = 6) with the sciatic nerve exposed but not ligated.

In order to assess mechanical allodynia, rats were placed in cages with an elevated metal mesh floor and given at least 30 min to acclimate. Mechanical allodynia was assessed by recording paw withdrawal in response to increasing stimulation with a series of calibrated von Frey filaments (Aesthesio, San Jose, CA, USA) ranging between 0.6 and 60 g in the ipsilateral paw’s medial plantar area. The 50% paw withdrawal threshold was determined using the method previously described by Dixon [41].

**Figure 1:**
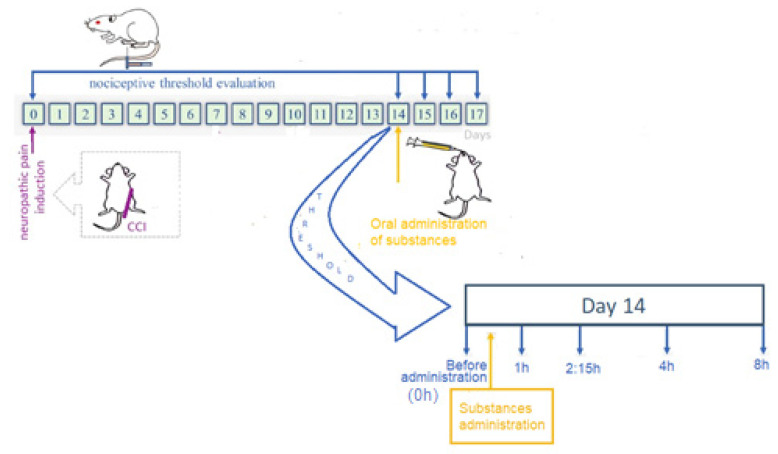
The experimental design was adopted to evaluate pregabalin delivery systems based on polyelectrolyte nanocomposites in comparison to the free drug for oral treatment of neuropathic pain. CCI—chronic constriction injury.

The nociceptive threshold was determined before the development of neuropathic pain (baseline latency–BL, on day 0). Thus, based on a previous investigation [35], mechanical allodynia was evaluated on the fourteenth day of CCI. On the fourteenth day, the drugs were delivered in a single dosage. In order to prevent interfering with absorption, animals were fasted for at least six hours before and up to two hours following drug administration. In order to determine the effect in hours, the nociceptive threshold was determined before and after drug administration at 1, 2:15, 4, 8, 24, 48, and 72 h (Figure 1). The measurements were always taken throughout the 12 h light cycle.

### 2.7. Motor Coordination and Balance Evaluation

The rats were split into four groups (n = 6): water (control), PG, PG-PN, and EMP-PN. A rotarod device (Insight, Ribeirão Preto, Brazil) was used to test motor coordination and balance. All rats were subjected to a two-day training session, during which time they achieved a consistent baseline level of performance [42]. Rats were trained to walk against the rotation of a revolving drum at a speed of 5 to 37 revolutions per minute (R.P.M.) for a maximum of 4 min (min) at that time. Following the training days, a one-day test was conducted by utilizing the apparatus’s accelerating speed level (5 to 37 R.P.M.) mode for four minutes. For the beginning, the baseline latency (BL) was determined, then new tests were conducted 1, 2:15, 4, 8, 24, and 48 h after the drugs were administered. The average time it took for the rotarod to fall off was then recorded.

### 2.8. Assessment of Barbiturate-Induced Sleep

The animals (n = 12 per group) were split into four groups: water (control); PG; PG-PN; and EMP-PN. They were administered thiopental (40 mg/kg IP) 1 h after receiving the drugs [35]. Anesthesia time was calculated as the time between the loss of straightening reflex and the time it would take for the reflex to return [43,44].

### 2.9. Statistical Analysis

In pharmacokinetic analysis, data are given as a median, and in behavior experiments, data are reported as a mean ± standard error of the mean (S.E.M.). The Mann–Whitney two-tailed test was used to analyze pharmacokinetic data. Two-way analysis of variance (ANOVA) with repeated measurements followed by a Bonferroni test was used to analyze mechanical allodynia. Motor test and barbiturate-induced sleep were compared using a two-way ANOVA with repeated measurements followed by a Newman–Keuls test. Statistical tests were conducted with a 95 percent confidence interval. Statistical tests were performed with Statistica 7.0 (StatSoft Power Solutions, Inc., Hamburg, Germany); the threshold of significance for all statistical tests was set at 5%.

## 3. Results

### 3.1. Characterization of PNs

**Figure 2:**
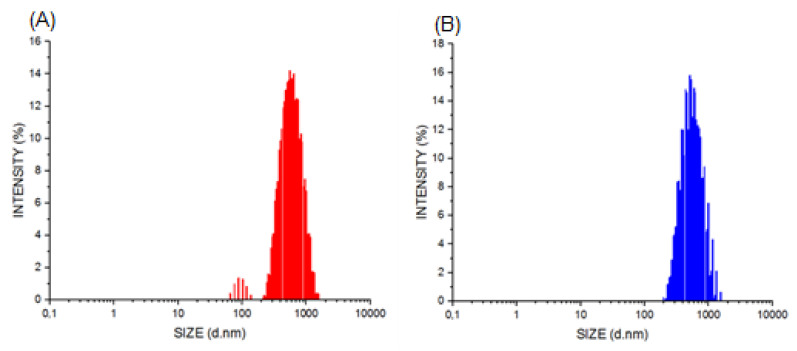
Distribution of PN’s size by intensity analyzed by DLS: EMP-PN (**A**) and PG-PN (**B**).

**Figure 3:**
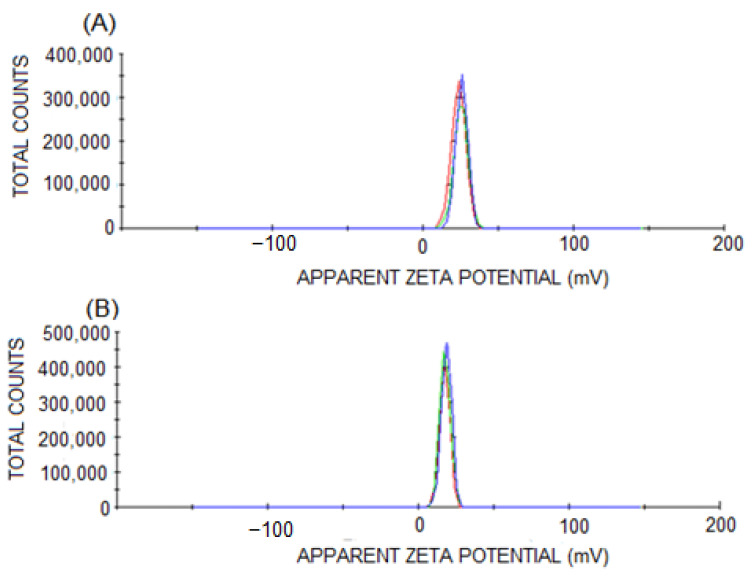
Distribution of PN’s zeta potential: EMP-PN (**A**) and PG-PN (**B**).

The PNs suspensions were homogenous and opalescent to a slight degree. The average particle size of PG-PN was determined using the DLS method, PdI, and the zeta potential of 432 ± 20 nm; 0.238 ± 0.001; and +19.0 ± 0.9 mV. For EMP-PN, these values are 425 ± 24 nm; 0.234 ± 0.016; and +18.6 ± 1.0 mV, respectively (Figure 2 and Figure 3).

PG-PN had a pH of 5.7 ± 0.06 while EMP-PN had a pH of 5.6 ± 0.08. Due to the fact that this drug is easily absorbed by oral route of administration in the pH range of 5.5–6.3 [45] and that it is required for the formulation’s equilibrium and stability, this pH of the PG-PN formulation can be considered adequate for oral PG absorption.

**Figure 4:**
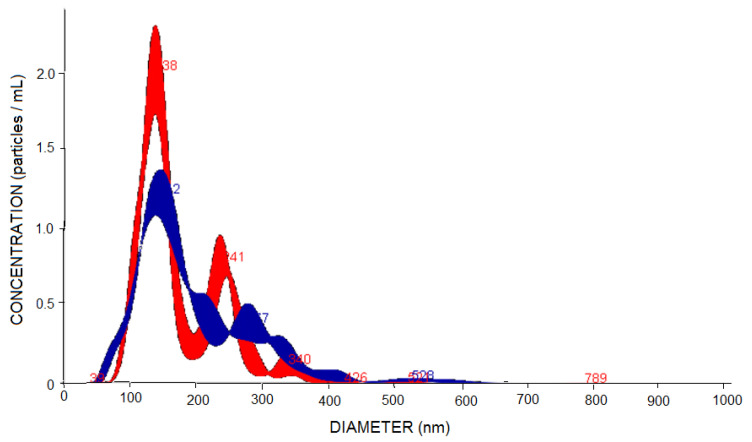
Distribution of PN’s size by concentration analyzed by NTA: EMP-PN (red) and PG-PN (blue).

The size distribution of PNs using the NTA method (Figure 4) is as follows: around 70% (EMP-PN or PG-PN) between 100 and 200 nm (average size: EMP-PN = 174 nm and PG-PN = 193 nm) and also between 200 and 300 nm and 300–500 nm (about 30 percent). These bigger size populations are compatible with the DLS results because the laser incidence angle is fixed in the latter approach; thus, the larger particles scatter lighter and are identified as a monodisperse population [46].

**Figure 5:**
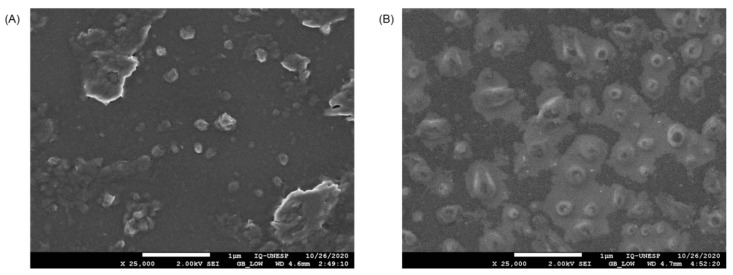
High-resolution field emission scanning electron microscopy (SEM) of PG-PN (**A**) and EMP-PN (**B**).

Figure 5 shows SEM pictures of PNs with rounded shapes and nanometric sizes, which correspond well with NTA and DLS results. Furthermore, due to the intumescence of the polymers in an aqueous medium, the EMP-PN particles have a lighter halo around them (Figure 5B), indicating that the particles are breaking apart after 30 days in the aqueous medium. This halo surrounding the EMP-PN was not observed in PG-PN (Figure 5A), suggesting that the particles become more stable when PG is added.

**Figure 6:**
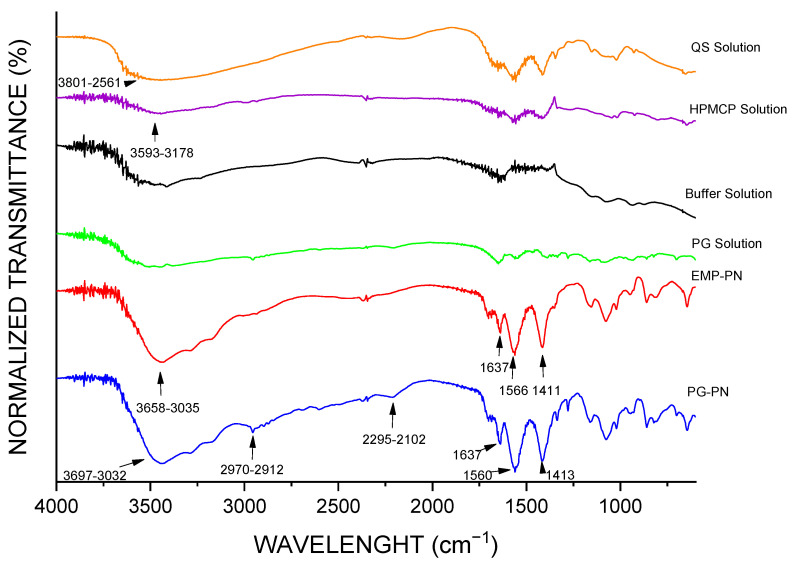
FT-IR by KBr dropping of nanocomplexes and formulation components.

**Figure 7:**
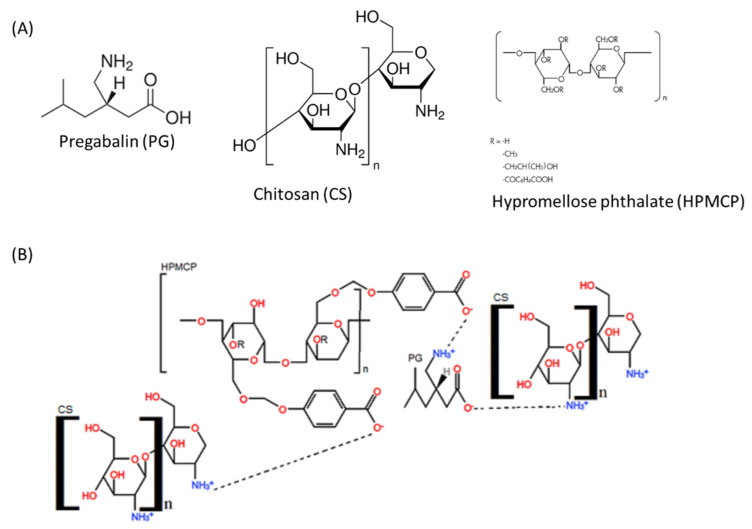
(**A**) Structural formula of pregabalin, chitosan, and hypromellose phthalate. (**B**) Possible interaction between the polymers and pregabalin.

The development of a deformation band at 1411 cm^−1^ (EMP-PN) and 1413 cm^−1^ (PG-PN) in the FT-IR spectra (Figure 6) suggested polyelectrolyte complexation since ammonium ion and carboxylate ion enhances absorption in this spectral region. Furthermore, there were more strong bands in the PG-PN and EMP-PN spectrums in the range of 1700–1500 cm^−1^ regarding the interacting carboxylate ion and ammonium ion, producing carboxylic acid and amine, as predicted. Furthermore, there was displacement and higher absorption intensity in the N-H stretching band overlapping the O-H stretching in the PG-PN and EMP-PN spectrums (3697–3032 cm^−1^ for PG-PN and 3658–3035 cm^−1^ for EMP-PN), which is consistent with polymer complexation. Furthermore, the presence of the most intense band in the PG-PN spectrum between 2970 and 2912 cm^−1^, which refers to the stretching of the CH_3_ group, shows that pregabalin interacts with the polymers and alters the conformation of the PN. Furthermore, a band between 2295 and 2102 cm^−1^ emerged in the PG-PN spectra, corresponding to NH_3_^+^ overtones, which indicates that PG or QS amine groups are more exposed as a result of the drug’s change in particle shape. Figure 7 depicts the structural formula of the isolated compounds as well as the potential interactions between the polymers and the polymers and PG.

**Figure 8:**
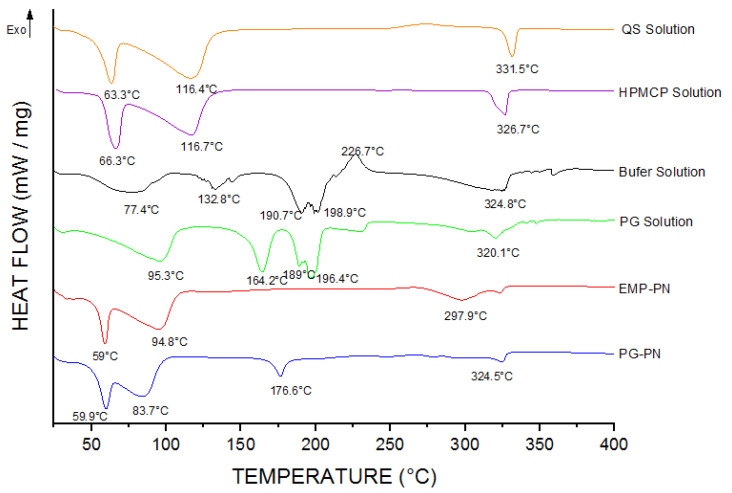
DSC curves of the polyelectrolytic nanocomposites and formulation components obtained in the heating range of 25–400 °C at a heat flow of 10 °C/min under dynamic nitrogen atmosphere flow (50 mL/min).

**Figure 9:**
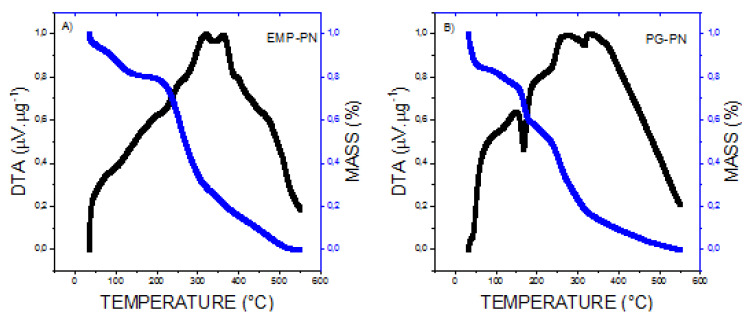
TG (in blue line) and DTA (in black line) curves of EMP-PN (**A**) and PG-PN (**B**) obtained due to heating at 10 °C/min in the range from 25 to 550 °C, under dynamic nitrogen atmosphere.

DSC (Figure 8) and TG/DTA (Figure 9) were also used to demonstrate nanocomposite formation. The presence of high-temperature events (297.9 °C and 324.5 °C, respectively), which are characteristics of thermal decomposition, suggest polymeric nanocomposite formation. While the endothermic events at 94.8 °C in EMP-PN and 83.7 °C in PG-PN correspond to the polymers, these peaks shifted relative to the solution of isolated polymers (116.4 °C for QS and 116.7 °C for HPMCP), which suggests nanocomposite formation. Furthermore, PG-PN differed from EMP-PN by exhibiting a peak corresponding to PG melting (176.6 °C), which was also displaced when compared to PG in the solution (164.2 °C), implying that the PG was not entirely integrated into the polymer matrix or did not fully interact with the polymers. Additionally, the peaks at 59 °C and 94.8 °C (EMP-PN) and 59.9 °C and 83.7 °C (PG-PN) indicated a glass transition with hysteresis followed by the release of water molecules from polymeric interstice.

According to the TG thermograms (Figure 9), the EMP-PN showed an initial mass loss of 20%, indicating dehydration, and a sudden mass loss from 195 °C, indicating breakdown of the polymeric matrix produced (Figure 9A). PG-PN showed an initial mass loss of 25%, implying dehydration, as well as a mass loss of 15% between 150 °C and 175 °C, indicating the decomposition of PG that did not interact with the polymeric matrix, as well as significant mass loss from 200 °C, denoting degradation of the polymeric matrix formed (Figure 9B). These findings are congruent with the DSC findings.

### 3.2. Pharmacokinetic Study

Table 1 shows that when the median of each pharmacokinetic parameter (AUC, Cmax, Tmax, Vd, t_1/2_, and clearance) was assessed, PG-PN had a significant decrease in AUC and Cmax (*p* < 0.05 and *p* < 0.01, respectively) compared to PG. Tmax and t_1/2_ of PG-PN were not different (*p* > 0.05) from PG. Furthermore, there was a substantial increase in Vd and the clearance of PG-PN (*p* < 0.05 for both parameters) than compared to PG. When compared to PG, PG-PN raised Vd by 3.3 times, showing that PG-PN allowed for higher PG distribution throughout the organism.

**Figure 10:**
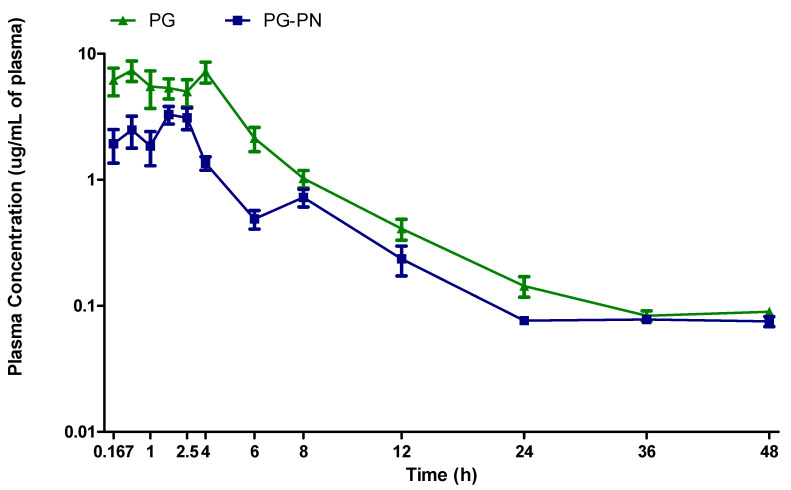
Mean pharmacokinetic profile after oral administration of PG and PG-PN (10 mg/kg) in rats (n = 6, for each profile) administered as a single dose.

Furthermore, a representative figure of mean values obtained in each non-compartmental profile demonstrates that PG achieved greater plasma concentrations than PG-PN (Figure 10). Figure 10 further showed that the release of PG from the PN happened in two stages. The first has a maximum peak time of one hour, and the second has a maximum peak time of eight hours. The first step is associated with the instantaneous release of PG that was not previously present in the polymer matrix, whereas the second stage is associated with the release of PG from the polymer matrix.

### 3.3. Antinociceptive Effect Study

**Figure 11:**
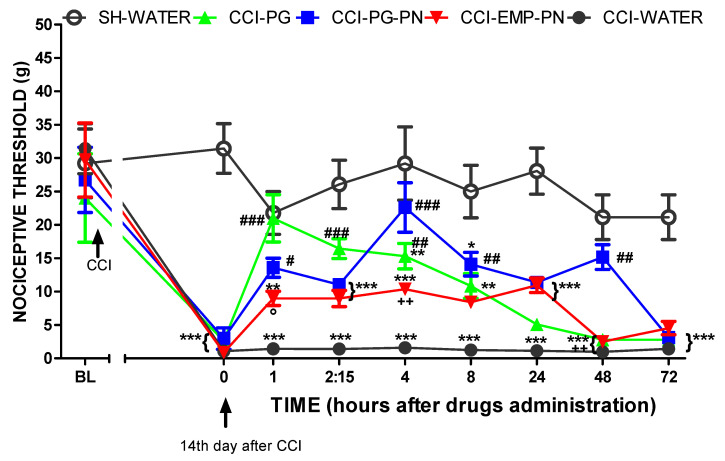
The antinociceptive impact of the drugs—operated animals and sham—compared 72 h after treatment. Legend: Pregabalin: PG (10 mg/kg, Orally); WATER: vehicle of PG; PN: polymeric nanocomposite; PG-PN: PG-loaded polymeric nanocomposite (10 mg PG/kg, Orally); EMP-PN: polymeric nanocomposite without PG (vehicle of PG-PN); CCI: chronic sciatic nerve constriction injury; SH: sham; BL: baseline latency; 0: on 14th day after CCI, immediately before administration of the substances; experimental time: 72 h after administration of the substances. Two-way ANOVA test with repeated measures with Bonferroni post-test was applied for the comparison between the different groups, * *p* < 0.05; ** *p* < 0.01; *** *p* < 0.001 when compared with the SH-WATER group, ## *p* < 0.01; ### *p* < 0.001 when compared with the CCI-WATER group; ++ *p* < 0.01 when compared with the CCI-PG-PN group; ° *p* < 0.05 when compared with the CCI-PG group. Each point represents the mean ± standard error of the mean (±S.E.M.) of six animals.

When compared to sham rats, CCI caused mechanical allodynia in all groups on the fourteenth day before drug administration (*p* < 0.01, F_4,25_ = 4.05), (Figure 11).

Figure 11 also shows that the animals with neuropathic pain pretreated with PG or PG-PN (CCI-PG or CCI-PG-PN) showed lower mechanical allodynia than the CCI-WATER group after 1, 2:15, and 4 h (*p* < 0.001, *p* < 0.001 and *p* < 0.01, F_4,25_ = 4.05, respectively) and 1, 4, 8, and 48 h (*p* < 0.05. *p* < 0.001, *p* < 0.01 and *p* < 0.01, F_4,25_ = 4.05, respectively) of its administration, respectively. In addition, this antinociceptive effect was longer (up to 48 h) in the PG-PN group compared to the PG group (*p* < 0.01).

Furthermore, the CCI-EMP-PN group showed no significant differences from the CCI-WATER group at any time, showing that EMP-PN had no antinociceptive impact.

However, the CCI-PG-PN group had a significantly higher antinociceptive impact than the CCI-PG, CCI-EMP-PN, and CCI-WATER groups at 48 h (*p* < 0.01, F_4,25_ = 4.05), indicating a sustained antinociceptive effect caused by pregabalin release from the polymeric matrix. Furthermore, when the CCI-PG and CCI-PG-PN groups were compared, the antinociceptive profile was similar as there were no significant differences, except 48 h after administration of the substances *p* < 0.01, F_4,25_ = 4.05), as previously mentioned, indicating that PG was released for a longer time.

The CCI-PG group only had a significantly greater antinociceptive effect than the CCI-EMP-PN group 1 h (*p* < 0.05, F_4,25_ = 4.05) after the substances were administered; the rest of the evaluations had a similar antinociceptive profile because there were no significant differences, indicating that EMP-PN has a similar antinociceptive effect potential to PG. Furthermore, when comparing the CCI-PG-PN groups with the CCI-EMP-PN groups, there was a significant difference only at times 4 h (*p* < 0.01, F_4,25_ = 4.05) and 48 h (*p* < 0.01, F_4,25_ = 4.05), which showed that EMP-PN has antinociceptive potential owing to the presence of chitosan.

**Figure 12:**
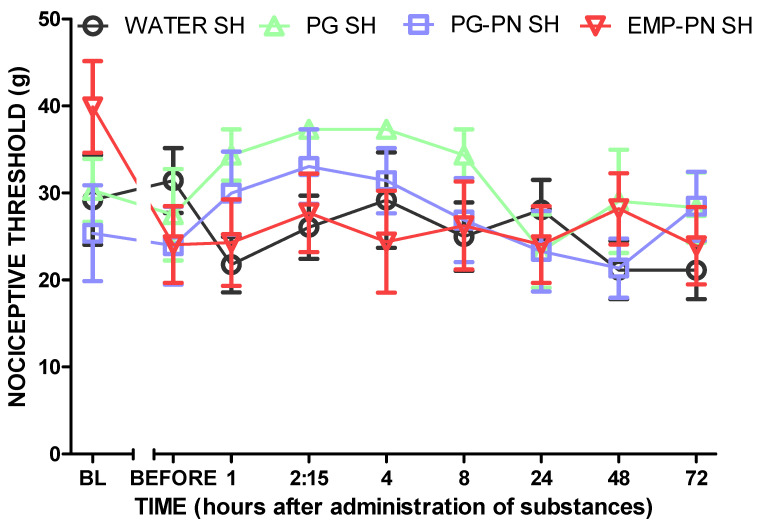
Antinociceptive effect of the comparison between the substances—sham animals—during 72 h after administration. Legend: Pregabalin: PG (10 mg/kg, Orally); WATER: vehicle of PG; PN: polymeric nanocomposite; PG-PN: PG-loaded polymeric nanocomposite (10 mg PG/kg, Orally); EMP-PN: polymeric nanocomposite without PG (vehicle of PG-PN); SH: sham; BL: baseline latency; BEFORE: on day 14 after CCI, immediately before administration of the substances; experimental time: 72 h after administration of the substances. A two-way ANOVA test with repeated measures with Bonferroni post-test was applied for the comparison between the different groups; there was no significant difference between the groups. Each point represents the mean ± standard error of the mean (±S.E.M.) of six animals.

Figure 12 shows that the nociceptive threshold did not change in the sham groups that received substances (*p* > 0.05, F_3,20_ = 0.91), suggesting that the sham procedure did not generate induced neuropathic pain and that the substances had no hypoalgesic impact.

### 3.4. Motor Coordination and Balance Evaluation

**Figure 13:**
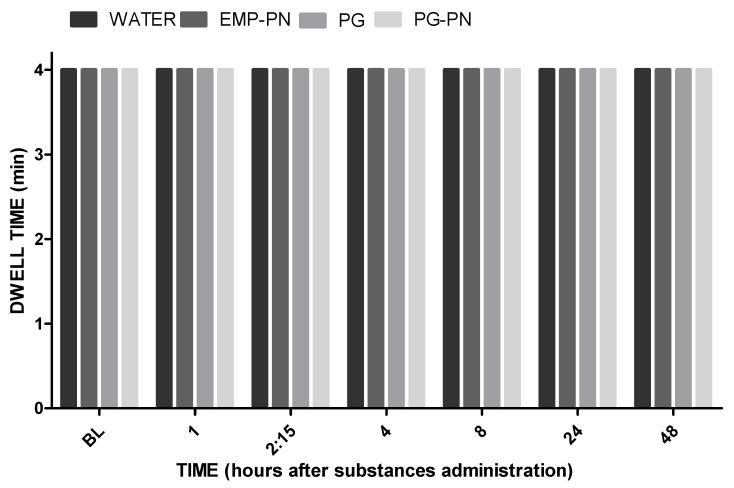
Motor coordination and balance evaluation. Legend: Pregabalin: PG (10 mg/kg, Orally); WATER: vehicle of PG; PN: polymeric nanocomposite; PG-PN: PG-loaded polymeric nanocomposite (10 mg PG/kg, Orally); EMP-PN: polymeric nanocomposite without PG (vehicle of PG-PN); BL: baseline latency; experimental time: 48 h. A two-way ANOVA test with repeated measures with Newman–Keuls post-test was applied for comparison between the different groups. No significant difference was observed. Each bar represents the mean ± standard error of the mean (±S.E.M.) of six animals.

According to Figure 13, all animals were able to maintain balance on the rotating bar for the established period (4 min) and at all tested times (BL, 1, 2:15, 4, 8, 24, and 48 h) (*p* > 0.05), demonstrating that the administration of the substances did not cause motor coordination loss in the animals and implying that the administered substances do not cause motor neurological deficit. Furthermore, this finding allows us to conclude that the antinociceptive impact of PG, PG-PN, and EMP-PN (item 3.2) was sensory rather than motor.

### 3.5. Evaluation of Barbiturate-Induced Sleep

**Figure 14:**
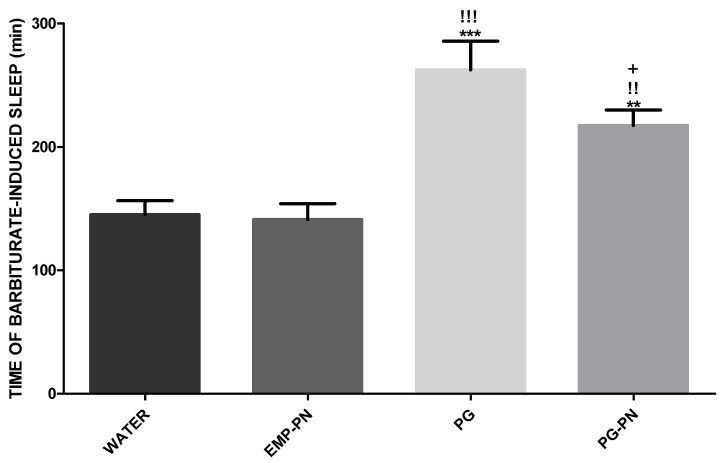
Evaluation of barbiturate-induced sleep time. Legend: Pregabalin: PG (10 mg/kg^−1^, Orally); WATER: vehicle of PG; PN: polymeric nanocomposite; PG-PN: PG-loaded polymeric nanocomposite (10 mg PG/kg, Orally); EMP-PN: polymeric nanocomposite without PG (vehicle of PG-PN). Administration of thiopental (40 mg/kg, I.P.) 1 h after the administration of substances for sleep induction. One-way ANOVA test with Newman–Keuls post-test was applied for comparison of the different groups, ** *p* < 0.01 and *** *p* < 0.001 when compared with the WATER group; !! *p* < 0.01 and !!! *p* < 0.001 when compared with the EMP-PN group; + *p* < 0.05 when compared with the PG group. Each bar represents the mean ± standard error of the mean (±S.E.M.) of 12 animals.

Figure 14 shows that the WATER and EMP-PN groups had the same sleep time since there was no significant difference (*p* > 0.05), indicating that the EMP-PN components (polymers) do not have nonspecific CNS depressive activity. Furthermore, when compared to the WATER group, the PG and PG-PN groups showed a significant increase in sleep time (*p* < 0.001 and *p* < 0.01, respectively), demonstrating that both substances have CNS depressant action because the barbiturate-induced sleep was potentiated 1.81 and 1.50 times, respectively, as expected and because the test principle states that substances with CNS depressant action, in general, reduce latency anesthesia [1].

Furthermore, when compared to the EMP-PN group, there was a significant increase in sleep time in the PG and PG-PN groups (*p* < 0.001 and *p* < 0.01, respectively), demonstrating that, in addition to PG and PG-PN being CNS depressants, the depressant action occurs only due to PG, as EMP-PN has the same sleep time as WATER.

The most significant outcome of this study was a 0.83-fold reduction in sleep time in the PG-PN group compared to the PG group (*p* < 0.05), demonstrating that when PG is delivered in a polymeric nanocomposite, its major adverse effect, which is drowsiness, is reduced.

## 4. Discussion

The lack of effectiveness and limitations of existing therapies, as well as side effects and tolerance to medicines, are known to render treating neuropathic pain challenging [47,48,49]. Pregabalin (PG) is the first-line therapy for neuropathic pain [50]. However, this drug’s adverse effects cause treatment abandonment [4,5]. With this in mind, we created a controlled release pregabalin system (PG-PN) and tested it in vivo.

For this, we considered the use of the natural polymers HPMCP and chitosan. Chitosan is depolarized by the action of lysozyme and N-acetylglucosaminase, generating the sugars N-acetylglucosamine and glucosamine, which explains its biodegradable characteristic. Due to the fact that it has low toxicity, chitosan can be considered biocompatible [51]. HPMCP is a cellulose derivative that is not absorbed by the body and has low toxicity, and it is also considered biocompatible and biodegradable [52]. In a previous study of the group [53], polymeric nanocomposites obtained by ionic cross-linking of chitosan and HPMCP presented low toxicity against fibroblast cells of the CCD-1059sk strain. For these reasons, polymeric nanocomposites of chitosan and HPMCP are considered safe for oral administration.

PG-PN and EMP-PN exhibited similar mean diameters, indicating that the medication did not appreciably alter PN size. Nanoparticles smaller than 500 nm can penetrate the blood–brain barrier and operate on the central nervous system [54,55]. As a result, these particles are smaller than other studies’ suggested microspheres containing PG [10,11,12]; therefore, they are suited for the intended goal.

The average PdI values obtained for PG-PN and EMP-PN were less than 0.250, which is below the maximum permissible limit for polymeric nanoparticles of 0.300. However, mini-emulsion eye drops containing PG [56] with a PdI smaller than 0.38 and the size distribution were also found to be homogeneous because we know that uncoiled polymer chains scatter light, affecting the overall size measurement [57,58].

Moreover, PG-PN and EMP-PN had similar zeta potentials (+20 mV). The polymers in the formulation determined the particle charge [59]. Due to the fact that sample pH (5.7) is lower than the chitosan pKa (6.5), the positive zeta potential is related to the chitosan contained in the polymer matrix [60]. The positive zeta potential in chitosan-covered mini-emulsion with PG [56] was observed. The zeta potential values of PG-PN were close to EMP-PN in our investigation, indicating that PG is entangled in the polymer matrix.

The pH of PG-PN was 5.7, and the pH EMP-PN was 5.6. The pH readings were not substantially different, showing that the presence of PG does not affect the formulation’s final pH. Moreover, the pH of the PG-PN formulations is suitable for oral PG absorption, since this medication is efficiently absorbed in a pH range of 5.5–6.3 [45].

Furthermore, spherical particles have more surface area than shapeless particles, which improves interaction with the intestinal epithelium and, hence, oral absorption [61]. The fact that the PNs in this investigation were spherical and nanosized implies that they are suitable for oral absorption and CNS activity.

FT-IR analysis revealed that PG crosslinks with QS and HPMCP polymers in an ammonium-carboxylate reaction. In thermal analysis, the absence of peaks corresponding to the drug’s melting point indicates that the drug is in amorphous form and, hence, molecularly dispersed in the polymer matrix [62,63]. Endothermic peaks around the melting of PG were observed in microspheres [12], showing that PG does not interact with mucilage. This is also observed in the present study. However, other studies noticed the absence of the endothermic peak associated with PG melting in the produced microspheres [10,11].

Pregabalin contains the same functional groups as chitosan (Figure 7A), making it difficult to separate these components and to determine encapsulation efficiency. However, the current study’s drug to polymer ratio is known to be the majority (3.18 parts PG: 3 parts QS: 1 part HPMCP, % *m/m/m*), suggesting that PG was not entirely absorbed into the polymer matrix. The percentage of PG that is not linked with the polymeric matrix, on the other hand, plays an essential role in reaching the initial therapeutic plasma concentration of PG.

The bioavailability and maximum plasma levels of PG-PN were found to be lower than PG following a single oral dosage. A strong ionic connection between PG and polymers may explain why PG is not completely released from the polymer matrix. Other investigations employing pregabalin-containing pharmaceuticals [6,20,64] found similar bioavailability and maximum plasma concentration reductions. Other investigations employing pregabalin-containing pharmaceuticals found improved bioavailability and maximum plasma levels [12,19].

The results suggest that PG-PN allowed for more extensive distributions of the substance and deposition in tissues and organs, modifying the Vd [65]. As the volume of distribution grows, so does clearance, as these two factors are proportional. Moreover, higher clearance reduces bioavailability [66]. In contrast, none of the authors that proposed modified-release pharmacological forms for PG published the estimated Vd and Cl values [6,10,11,12,20,64].

Oral PG-PN and PG were used in this work to alleviate CCI-induced neuropathic pain in rats. As predicted, PG and PG-PN had antinociceptive effects, as PG has been demonstrated to decrease mechanical allodynia in rats [34,67,68,69,70]. PG-PN had a longer profile of action than PG, lasting up to 48 h after administration, whereas PG only lasted up to 4 h. The current study’s 4 h impact is in line with another study that showed an antinociceptive effect of pregabalin for 8 h [70]. Thus, customized release systems such as microspheres and polymeric nanocomposites are efficient in delaying the release of the active [10]. An intranasal PG microsphere demonstrated better anticonvulsant efficacy than PG given peripherally [11], indicating that modified-release pharmacological formulations of PG are superior to traditional therapy.

The analgesic action of chitosan is due to the absorption of proton ions by ionization of the amino group to NH_3_^+^ [71]. Furthermore, because pregabalin and chitosan have the same functional groups at Figure 7A, we hypothesized that chitosan acts on the same therapeutic targets as pregabalin (voltage-dependent calcium channels) with respect to exerting the antinociceptive effect, but there is no evidence in the literature to support this hypothesis. However, EMP-PN’s antinociceptive effect is weaker than the sham control animals’ (which received water). The fact that EMP-PN is antinociceptive is beneficial since it permits the PG-PN vehicle to reduce nociception.

However, the results show that EMP-PN has an antinociceptive impact, whereas PG-PN has a synergistic antinociceptive effect of PG and chitosan. More research is needed to confirm synergistic impacts (additive or potentiating).

Furthermore, as no animals died or showed signs of intoxication after receiving EMP-PN or PG-PN, it appears that the formulation is safe at this dose and for the time observed, although more research is required [72].

In our study, rats were given one oral dosage of PG-PN or PG: Neither drug affected motor behavior nor caused motor impairments. Other researchers found the same PG motor coordination profile [70,73], proving that PG does not impair animal motor coordination. Thus, the efficiency of PG-PN in decreasing one of PG’s adverse effects in people, which is the loss of motor coordination, could not be evaluated [1].

PG-PN and PG were also examined in rats following a single oral dosage in order to measure sleepiness reduction [1]. Drowsiness was reduced in our study by 0.83 times in rats receiving PG-PN compared to animals receiving PG, which supports another study [31].

The open-field test was also performed to examine the drugs’ sedative effects (data not displayed due to lack of impact). Upon re-exposure to the test, the animals displayed a profile of indifference in exploration that was independent of the drug provided, preventing the detection of CNS depressive impact of PG or PG-PN.

## 5. Conclusions

The present study used natural polymeric nanocomposites (PN) containing pregabalin (PG) for oral administration in order to minimize administration frequency and adverse effects. The polymeric nanocomposites showed acceptable physicochemical properties for prolonged PG release, making them suitable for in vivo testing. Due to the fact that the in vivo formulation has extended circulation, it has a longer profile of action. In animal trials, PG-PN increased the antinociceptive efficacy of PG in solution while reducing sleepiness, the drug’s major adverse effect. The oral administration of PG via natural polymeric nanocomposites may improve treatment compliance and patient well-being when used in clinical practice since it allows less frequent administration with fewer side effects.

## Figures and Tables

**Table 1:** Estimated pharmacokinetic parameters after oral administration of PG and PG-PN (10 mg/kg) in rats (n = 6, for each profile). Data expressed as median, CI = 95%.

Parameter	PG	PG-PN
AUC (^0-∞^) (µg/mL h)	39.60 (82.55–24.25)	22.08 * (32.16–13.53)
Cmax (µg/mL)	6.56 (5.06–13.88)	3.48 ** (5.70–1.40)
Tmax (h)	0.75 (0.5–4)	1.5 (0.5–2.5)
Vd (L/kg)	2.51 (1.82–6.44)	8.21 * (98.02–3.33)
t_1/2_ (h)	7.03 (6.68–10.83)	10.01 (178.35–7.42)
*Cl* (L/kg/h)	0.25 (0.12–0.41)	0.46 * (0.31–0.74)

Legend: Mann–Whitney test two-tailed. * *p* < 0.05, ** *p* < 0.01.

## Data Availability

The data presented in this study are available on request from the corresponding author. The data are not publicly available once we can keep these data confidential, as per university policy, until the publication of articles.
